# Phase Behaviour and Miscibility Studies of Collagen/Silk Fibroin Macromolecular System in Dilute Solutions and Solid State

**DOI:** 10.3390/molecules22081368

**Published:** 2017-08-18

**Authors:** Ima Ghaeli, Mariana A. de Moraes, Marisa M. Beppu, Katarzyna Lewandowska, Alina Sionkowska, Frederico Ferreira-da-Silva, Maria P. Ferraz, Fernando J. Monteiro

**Affiliations:** 1 i3S—Instituto de Investigação e Inovação em Saúde, Universidade do Porto, Rua Alfredo Allen, 208, 4200-135 Porto, Portugal; ffsilva@ibmc.up.pt; 2 INEB—Instituto de Engenharia Biomédica, Universidade do Porto, Rua Alfredo Allen, 208, 4200-135 Porto, Portugal; 3 FEUP, Faculdade de Engenharia, Departmento de Enginharia Metalurgia e Materiais, Universidade do Porto, 4200-465 Porto, Portugal; 4 School of Chemical Engineering, University of Campinas, 13083-852 Campinas, Brazil; mamoraes@unifesp.br (M.A.d.M.); beppu@feq.unicamp.br (M.M.B.); 5 Department of Chemical Engineering, Federal University of São Paulo, 09913-030 Diadema, Brazil; 6 Nicolaus Copernicus University in Toruń, Faculty of Chemistry, Department of Chemistry of Biomaterials and Cosmetics, ul. Gagarina 7, 87-100 Toruń, Poland; reol@chem.umk.pl (K.L.); as@chem.umk.pl (A.S.); 7 IBMC—Instituto de Biologia Molecular e Celular, Universidade do Porto, Rua Alfredo Allen, 208, 4200-135 Porto, Portugal; 8 FP-ENAS/CEBIMED, University Fernando Pessoa Energy, Environment and Health Research Unit/Biomedical Research Center, 200-150 Porto, Portugal; mpferraz@ufp.edu.pt

**Keywords:** biopolymers, protein-protein interaction, silk fibroin, miscibility, coacervation

## Abstract

Miscibility is an important issue in biopolymer blends for analysis of the behavior of polymer pairs through the detection of phase separation and improvement of the mechanical and physical properties of the blend. This study presents the formulation of a stable and one-phase mixture of collagen and regenerated silk fibroin (RSF), with the highest miscibility ratio between these two macromolecules, through inducing electrostatic interactions, using salt ions. For this aim, a ternary phase diagram was experimentally built for the mixtures, based on observations of phase behavior of blend solutions with various ratios. The miscibility behavior of the blend solutions in the miscible zones of the phase diagram was confirmed quantitatively by viscosimetric measurements. Assessing the effects of biopolymer mixing ratio and salt ions, before and after dialysis of blend solutions, revealed the importance of ion-specific interactions in the formation of coacervate-based materials containing collagen and RSF blends that can be used in pharmaceutical, drug delivery, and biomedical applications. Moreover, the conformational change of silk fibroin from random coil to beta sheet, in solution and in the final solid films, was detected by circular dichroism (CD) and Fourier transform infrared spectroscopy (FTIR), respectively. Scanning electron microscopy (SEM) exhibited alterations of surface morphology for the biocomposite films with different ratios. Surface contact angle measurement illustrated different hydrophobic properties for the blended film surfaces. Differential scanning calorimetry (DSC) showed that the formation of the beta sheet structure of silk fibroin enhances the thermal stability of the final blend films. Therefore, the novel method presented in this study resulted in the formation of biocomposite films whose physico-chemical properties can be tuned by silk fibroin conformational changes by applying different component mixing ratios.

## 1. Introduction

Miscible blending of two biopolymers with different physicochemical characteristics is an interesting route to produce new materials with unique properties that may present the advantages of each single polymer and compensate the disadvantages over each one. The films prepared from blends of natural polymers can potentially be used in wound healing and skin tissue engineering applications [1,2]. The presence of proteins as natural macromolecules in blends may improve cell adhesion, due to the presence of more protein binding sites [3]. However, native physical structures of proteins, such as collagen, with a linear triple helix, limits its possible intra- and interchain interactions in blends [4,5]. The forces found in protein interactions are electrostatic, Van der Waals, hydrogen bonds, and hydrophobic and steric interactions, of which the electrostatic interactions are predominant [6]. Parameters such as pH and ionic strength may affect electrostatic interactions, whereas temperature may have an influence on hydrophobic and hydrogen bindings [7,8]. However, temperature induces protein denaturation. For example, heating collagen induces the cleavage of the intermolecular hydrophobic and hydrogen bonds, transforming the collagen triple helix into a randomly coiled form, allowing fibril formation and interactions with other proteins [9]. This work has been focused on the blending of collagen and silk fibroin as two relevant biomaterials showing high potential to be used for production of protein-based biocomposite films.

Collagen, as a biomaterial, is a key player in biomedical applications. Collagen acts as a natural scaffold for cell proliferation and has adequate mechanical strength, good biocompatibility, biodegradability, and ability to promote cellular attachment and growth. In addition, different functional groups along the collagen backbone may promote the incorporation of growth factors and other biological molecules [10]. Preservation of collagen native structure in biomedical applications may be of importance, since the collagen triple helix network may withstand the mechanical stresses through transmitting the forces and dissipating energy [11]. Moreover, the triple helix characteristics, such as high stability in the biological environment, binding ligands for cell surface receptors, and essential signals to influence cell activity [12,13], highlight the importance of protecting such conformational integrity for functional applications.

On the other hand, silk fibroin presents several interesting properties, such as excellent toughness and stiffness combined with low density. The high mechanical strength of silk fibroin attracted the attention of several researchers. Even though silk fibroin has good mechanical properties, the biomedical applications require other desired properties, such as high water retention capability and biodegradability, which are absent in the native silk fibroin. Hence, it is adequate to increase the amount of amorphous structure of fibroin molecules by dissolving fibers, disrupting the hydrogen interactions and inducing the transitions of fibroin to random coil conformation that results in regenerated silk fibroin solution (RSF) [14]. Solubility of native silk fibroin depends on the organic salts, which participate in the disruption of hydrogen bonds. Foo et al. [15], revealed that the hydrophilic parts of silk fibroin, stabilized by Ca^2+^ or other ions, cause the aggregation of fibroin molecules into hydrophilic regions, forming a gel through ionic crosslinks. Water molecules absorbed by hydrophilic regions restrain the premature crystallization of the hydrophobic domains. The highly concentrated salt solutions of silk fibroin have to be dialyzed in order to remove the salts and make it adequate for the preparation of SF-based materials [16].

The mechanism of fibroin self-assembly during dialysis has been described by several researchers [17,18], and the influence of various parameters, such as concentration [19], temperature [20], and ethanol content [21] on RSF self-assembly have been analyzed. Jin et al. [17] suggested a micellar structure pattern for silk fibroin chains in water in which the small hydrophilic parts of silk fibroin remain hydrated, while the large termini hydrophilic parts are located at the outer edge of micelles and the hydrophobic parts are placed between those two hydrophilic blocks [17]. Hence, the RSF solution after dialysis is water-soluble and metastable until the hydrophobic parts of micelles bind and eventually form gels. Even though RSF is a promising material with specific biological and functional characteristics, its partially amorphous structure, along with its limited solubility, restrain the applications of this biomaterial. Hence, blending with other biomaterials is a useful way to improve the properties of RSF [19].

Several studies on collagen/silk fibroin blends showed not only the improvement in mechanical properties of the final materials, but also a favorable environment of the mixtures for cell attachment and proliferation. Nevertheless, the blends were restricted to low collagen concentration or COL/RSF ratios, or to be able to incorporate higher collagen ratios, high temperatures are required that denature the collagen triple helix and facilitate the hydrogen bonding between the two biopolymers [21,22,23,24,25,26,27]. However, inducing electrostatic interactions between these two biopolymers, which has not been used up to now, may avoid the risk of the undesirable denaturation of collagen as the result of increasing the temperature. Aiming at preserving the collagen natural structure, this study tries to present a new method based on electrostatic interactions, for templating protein-protein interactions between collagen and regenerated silk fibroin macromolecules, in dilute solutions and in solid thin films. Considering the importance of salt for electrostatic interactions, three single-phase compositions of COL/RSF, with 75/25, 50/50 and 25/75 volume ratios (*v*/*v*), were prepared according to their phase diagram and dialyzed against distilled water. Different blends were obtained and physico-chemically characterized after dialysis.

## 2. Results

### 2.1. Miscibility Study of Collagen/Silk Fibroin

In this study, ternary solvent containing salt ions was used in order to induce electrostatic interactions between collagen and RSF chains and obtain miscible or semi-miscible blends.

**Figure 1:**
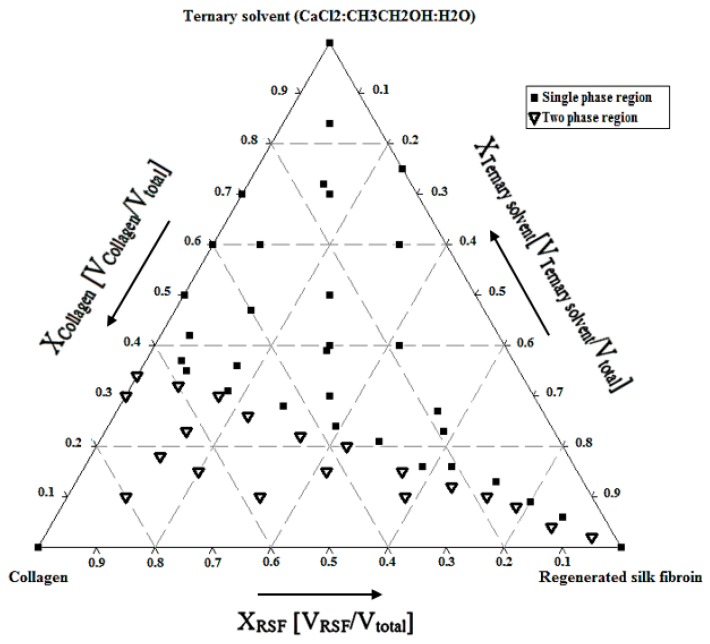
Ternary phase diagram of collagen/RSF/ternary solvent at 4 °C.

The borderline mixing ratio points of collagen/RSF/ternary solvent, were obtained by blending the different volumes of component, and the points were plotted in the ternary phase diagram as shown in Figure 1. The miscible solutions can be identified in the single phase region, while phase-separated solutions (which can be easily visually observed as the fibrils start to be formed) are indicated as the two phase region.

Regarding the polyelectrolyte nature of the involving proteins in this research, the electrostatic interaction between cationic amino groups of collagen and anionic groups of silk fibroin, and high calcium content, are the main cause of polyelectrolyte complex (PEC) formation.

The ζ potential measurements brought detailed understanding into the composition of charged groups in the mixture blends. Therefore, solutions with different mixing ratios according to the single-phase region in ternary phase diagram were measured and the results are shown in Table 1.

The net charge of all blends in the single phage region, is around zero, corresponding to almost electroneutrality of the solutions due to the high amount of salt. As shown in Table 1, the amount of ζ potential is negative for solutions containing more silk fibroin, which is attributed to higher amounts of silk chains with negative groups.

The quantitative analysis of miscibility by viscosimetry has been conducted by calculating the miscibility parameter (∆b_m_) ,as well as relative and reduced viscosities, both theoretically (by methods of Krigbaum and Wall [28], and Garcia et al. [29]), and experimentally, and plotted against solution concentration.

The theoretical and experimental values for pure collagen, pure silk fibroin and their blends, according to the single-phase region of ternary phase diagram, are shown in Table 2. The positive miscibility parameter for all the blends indicates good miscibility for all prepared blends, which is due to the electrostatic interactions between chains and calcium ions, making the whole mixture more stable.

In a highly soluble polyelectrolyte mixture containing high amount of salt, the individual chains of each polyelectrolyte are separated from each other, with salt ions placed among them that yields to the rising of solution viscosity [30]. Increasing salt concentration in the complex system leads to the screening of the electrostatic interactions between two macromolecules, as well as the rearrangement of the polymer chains that may raise the viscosity of solution [31].

**Figure 2:**
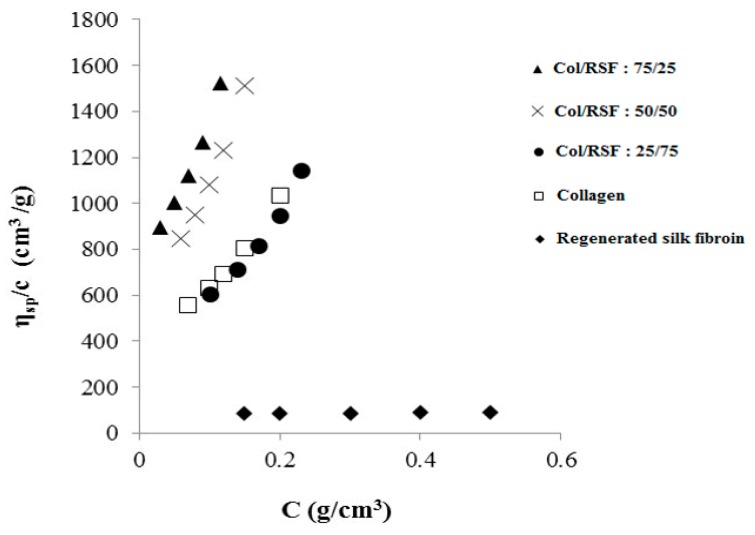
Reduced viscosity versus concentrations of collagen/RSF solutions.

Figure 2 shows the reduced viscosity versus the concentration for pure collagen, pure silk fibroin and their blends. Silk fibroin solution has the lowest viscosity of all the solutions due to the solvation of fibroin chains by calcium ions of the ternary solvent. The viscosity of the primary mixtures with 25%, 50% and 75% of collagen (without adding the dilution solvent), is higher than the viscosity of pure silk fibroin and pure collagen.

Mixtures with more collagen content have higher ionic strength and viscosity. Therefore, initial mixtures with 75% and 50% collagen were more viscous than the others. Additionally, the reduced viscosities of mixtures with 50% and 75% collagen show close values, both above the values for other solutions, illustrating the high ionic strength in these solutions.

However, adding NaCl solution as the diluting solvent to the aqueous system of proteins containing high calcium ions increases the probability of gradual precipitation after second time dilution. The imbalance of salt concentration among protein chains and the outside medium through dilution causes the calcium diffusion towards the medium by osmotic pressure. Moreover, the possibility of Ca^2+^ ion displacement by Na^+^, in the sites requiring the ions in a dehydrated state, alters the conformation of binding sites, which is due to the kosmotropic behavior of NaCl, together with the prevention of binding sites by calcium ions [32]. In view of these considerations, the precipitation caused by dilution can be controlled by careful selection of the added volume of the NaCl solution.

### 2.2. Phase Change after Collagen/Silk Fibroin Dialysis

Slow diffusion of calcium salt through dialysis procedure, during three days, changes the phase behavior of mixtures from a homogeneous solution to a coacervate or a precipitate, depending on the degree of neutralization. The phase behavior after the last day of dialysis, when only residual salt amount may be present, shows a solid-liquid phase separation with formation of white solid complex coacervates or precipitates, for all the mixtures, which could be easily identified by naked-eye and optical microscope. This solid-liquid phase separation occurs because the removal of salt ions through the dialysis membrane increases the interaction of proteins COO^−^ and NH^3+^ ionic groups.

**Figure 3:**
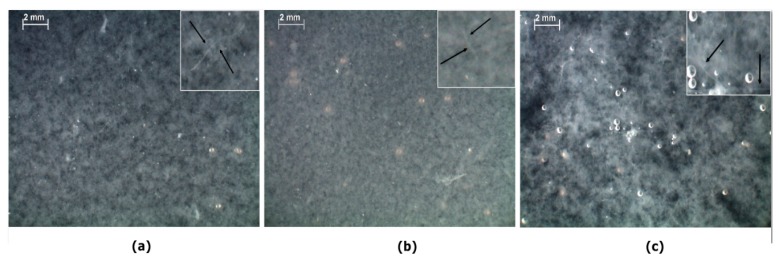
Optical microscope images of blend solutions after dialysis with the starting ratios (before dialysis) of: (**a**) Col/RSF: 75/25; (**b**) Col/RSF: 50/50; and (**c**) Col/RSF: 25/75. Arrows indicates fibril formation in the system.

Figure 3 shows optical stereoscopic microscope images of blend mixtures after dialysis for three days. It should be noted that all the mentioned ratios correspond to those before dialysis procedure. As shown in Figure 3, the aggregates size increases with increasing amount of silk fibroin, while fibril formation occurred in all the mixtures.

To study the effect of dialysis (salt removal) on coacervate complexes, ζ potential has been used, and the results for the solutions after dialysis are presented in Table 3. The results show that dialysis procedure increased the charge density of solutions, leading to stronger attractive interactions among oppositely charged polyelectrolytes. The positive charge density for all the mixtures indicates the excess of NH^3+^ groups, which increases with increasing the collagen ratio. However, the ζ potential for silk fibroin rich mixture is lower than the other ones, which can be related to higher amounts of silk fibroin negative charges in the mixture.

The ζ potential results are in agreement with optical images (Figure 3), proving that blend solutions of 75% and 50% collagen with high charge densities (Figure 3a,b), contain aggregates possessing charge-charge repulsion which inhibits their assembly. However, a lower charge density of solution with a higher silk fibroin ratio yields less repulsion and more aggregate assembly due to hydrophobic interactions (Figure 3c).

Finally, in order to confirm that the coacervate particles contain the complex of proteins, all the mixtures after dialysis procedure (as shown in Figure 3) have been passed through the gas and vacuum filters. Thereafter, the remaining solutions after filtration have been passed through two calibrated markers of the Ubbelhode viscosimeter, and the time has been measured. The RSF solution after dialysis with the reduced concentration from 0.5% to 0.17%, due to the dilution during three days of dialysis, showed a passing time of 111.16 s (without any filtration). Table 4 shows that, during three days of dialysis, all the aqueous solutions remaining after filtration present a passing time s close to that of distilled water, illustrating that the coacervate particles remaining behind the filters were protein complexes.

Slightly higher values of solution passing time than that of water may be due to the presence of remaining calcium ions in solution after interaction of collagen/RSF. Studies on the effect of lithium ions on silk fibroin films [33] confirmed that even after 72 days of dialysis, the salt ions could not be removed completely. The passing time is higher for the first day and decreases on the second and third days, respectively. After release of high salt levels during the first day, the gradual release during the second and third days is a combination of concentration gradient and the result of gradual electrostatic and hydrophobic interactions between the two polymers as well as the fibroin fibrillation. Due to the not-fully neutralized coacervates, these calcium ions may be electrostatically weakly bonded to the oppositely-charged free residues of the proteins inside the dialysis tube and, therefore, have more tendency to stay inside the dialysis tube rather than being removed to the water bath. However, more detailed studies are required to assess the amount of salt and non-mixed proteins in the remaining solutions after filtration.

### 2.3. Structural Characteristics of Collagen/Silk Fibroin Blend Solutions after Dialysis and Solid Films

After preparation of the blended films through drying of solutions at room conditions, their structures were analyzed via SEM, FTIR, DSC, and contact angle.

**Figure 4:**
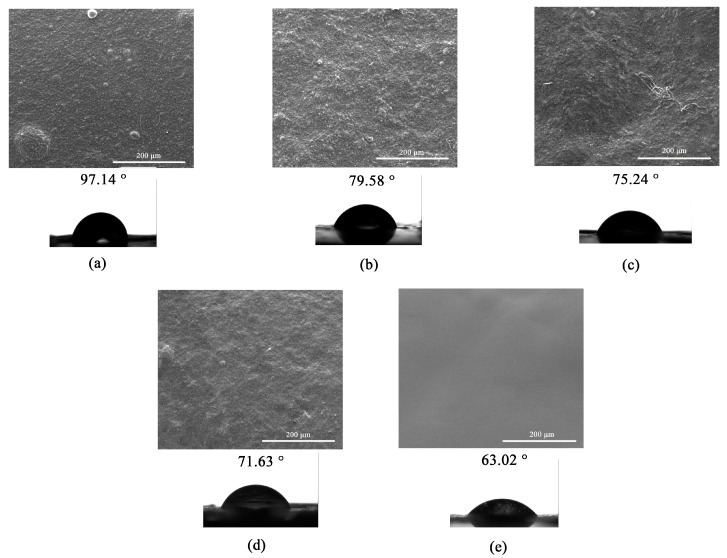
Scanning electron microscopy (SEM) and water contact angle images of blended films: (**a**) Collagen; (**b**) Col/RSF: 75/25; (**c**) Col/RSF: 50/50; (**d**) Col/RSF: 25/75; and (**e**) RSF at 500× magnification.

As can be seen in Figure 4, SEM images show a rough surface for collagen (Figure 4a) while a smooth surface for RSF (Figure 4e). According to the Col/SF ratios, significant changes in film surface morphology could be observed upon mixing RSF with collagen. Blended film with more collagen (Figure 4b) showed a surface similar to the collagen film (Figure 4a). However, the surface of the blended film with more silk fibroin (Figure 4d) has less roughness than the other ones. The fibrous-like structure could be observed for Col/RSF: 50/50 (Figure 4c), which confirms the previous results of optical microscopy (Figure 3).

Moreover, the contact angle measurements of the films’ surfaces shown in Figure 4 revealed that the RSF film had the highest hydrophilicity, and the hydrophilicity of blended films were in the range between pure silk fibroin and pure collagen. The results indicated that the hydrophilicity of the blended films improved with increasing the regenerated silk fibroin proportions. The observation demonstrated that the regenerated silk fibroin has significant influence on the wettability of the blended surfaces, which can be explained by the entanglement of RSF molecules and the exposure of their hydrophilic groups, which may be arranged on the surface of RSF chains [34].

**Figure 5:**
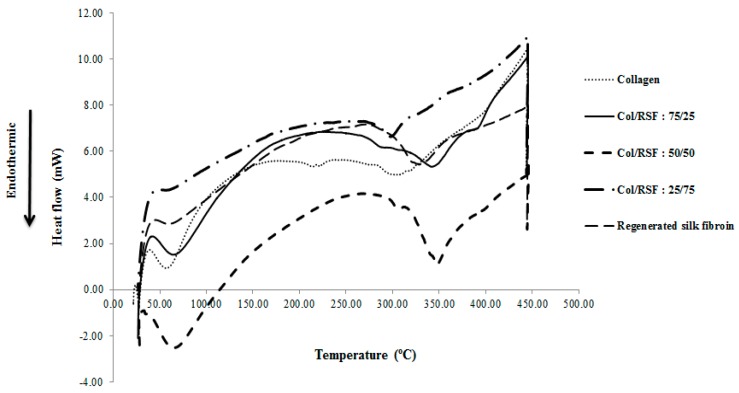
DSC curve of collagen/RSF blend films.

Figure 5 present the DSC curves of the prepared films. Collagen presents an endothermic peak at around 57 °C, attributed to the evaporation of unbounded water molecules and the denaturation of collagen fibrils [35]. However, the very small endothermic peak at around 308 °C corresponds to the breaking of hydrogen bonds between alpha chains and to collagen transformation from the triple helix to the random coil structure [36]. Regenerated silk fibroin presents a tiny endothermal peak at around 62 °C, which is related to the loss of unbound water molecules. The second endothermal peak is at around 330 °C, attributed to the thermal degradation of silk fibroin chains.

The absence of denaturation peak in the blend film containing higher silk fibroin ratio, shows the persistence of silk fibroin beta sheet structure in protection of collagen from denaturation through presumable covering of collagen chains while limiting the space for collagen triple helix motion. However, the existence of denaturation peak for other mixtures indicated that the collagen triple helix structure is maintained in those blend films. Thermal denaturation peaks in such blends shifted towards higher temperatures (65–67 °C) compared to collagen samples, demonstrating higher stability of the collagen triple helix. Thermal degradation of collagen/RSF mixtures with ratios of 75/25, 50/50, and 25/75 occurred at 342 °C, 348 °C (sharp peak), and 298 °C (small peak), respectively. The sample with higher silk fibroin ratio shows a tiny endothermic peak which is attributed to the molecular motions of alpha helix chains within the small amorphous regions. However, the absence of degradation peaks illustrates dominating silk fibroin beta sheet conformation. Nevertheless, mixtures with 50% and 75% collagen, show larger decomposition peak at higher temperatures, indicating that thermal decomposition is the sum of heat adsorbed to degrade the hydrogen bonds in both the collagen triple helix and the beta sheet structure of silk fibroin. In addition to the decomposition at higher temperature for the mixture with 50% collagen, the large observed decomposition peak may be the proof of the existence of more amorphous regions with alpha helix structures. Overall, decomposition of mixtures at higher temperatures than that of silk fibroin can be due to the beta sheet structures of silk fibroin in the blend films, along with higher thermal stability.

**Figure 6:**
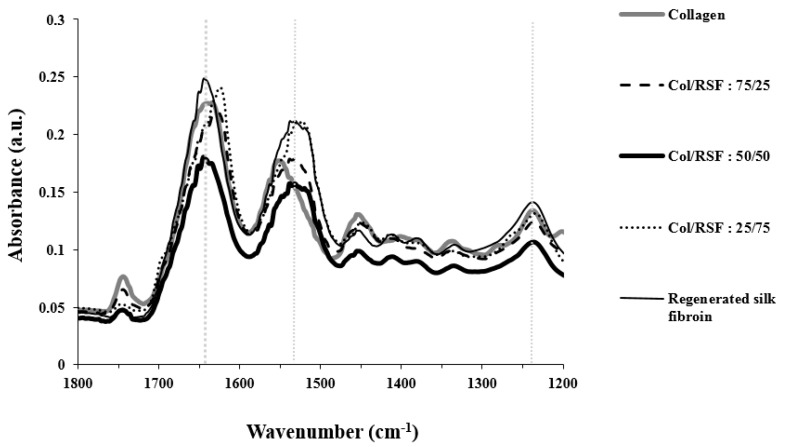
FTIR spectra of collagen/RSF films.

Figure 6 shows FTIR spectra of collagen/RSF blend films, as well as the individual polymer films. As reported in the literature, the spectral properties of silk fibroin showed two different structures of silk I and silk II, which are known to be rich in helical and beta-sheets, respectively. The spectral ranges of amide I (C=O and C–N stretching), amide II (N–H bending), and amide III (C–N stretching) are reported as 1655–1660 cm^−1^, 1531–1542 cm^−1^, and 1230 cm^−1^ for silk I, 1620–1630 cm^−1^, 1515–1530 cm^−1^ and 1240 cm^−1^ for silk II, and 1640–1648 cm^−1^, 1535–1545 cm^−1^, and 1235 cm^−1^ for random coil structures [37,38]. Collagen amide I has been separated into three component peaks, including 1628–1633 cm^−1^ for hydroxyproline [39]. Additionally, the amide II bands [40] are presented at around 1550 cm^−1^, and amide III bands [41] at around 1240 cm^−1^.

Amide I is the important peak in characterizing conformational changes. As shown in Figure 6, the position of amide I peaks of sample with 50/50 ratio remained unchanged for the fibroin molecules in the amide I region, showing the predominant contribution of silk fibroin with random coil chains in the mixture. Increasing collagen ratio to 75%, induced silk fibroin β-sheet structure as the amide I peak shifted to 1626 cm^−1^. However, the unchanged position of amide II at 1531 cm^−1^ is consistent with the existence of some alpha helix structures of silk fibroin in the mixture. Blends with higher amounts of silk fibroin showed the amide I peak at 1623 cm^−1^, while the amide II peak appeared near 1525 cm^−1^, indicating that the blended films contain mostly crystalline beta sheets that may be attributed to the fibrillogenesis of silk fibroin. Table 5, summarizes the assignment of the major IR peaks for each polymer and their blend films.

**Figure 7:**
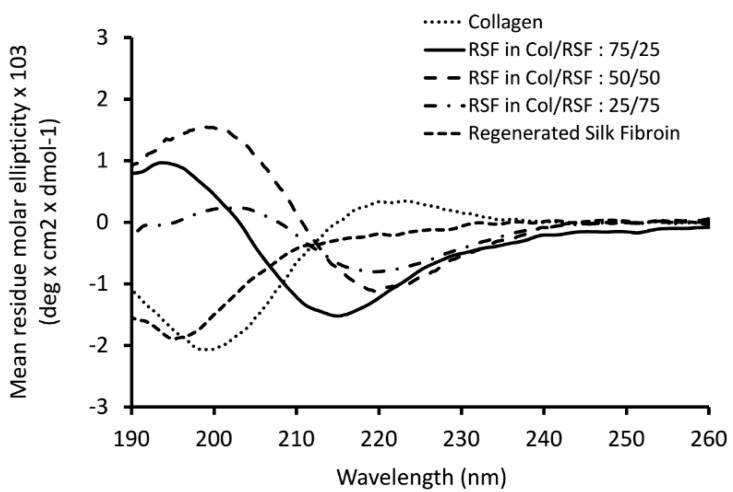
Circular dichroism (CD) spectrum of collagen/RSF mixtures after dialysis.

Circular dichroism (CD) analysis was performed in order to investigate and affirm conformational transition of silk fibroin in the blended solutions after dialysis. As shown in Figure 7, CD spectrum of pure silk fibroin after dialysis showed typical random coil structure with a negative peak near 195 nm. For pure collagen, it was observed triple helix characteristic spectrum with a large negative peak near 197 nm, as well as a small positive peak centered at 220 nm.

Considering the persistence of natural collagen structure in the mixtures that was confirmed by DSC analysis and in order to obtain the spectra for regenerated silk fibroin when blended with collagen, the spectrum of the later was subtracted from the spectra measured for the blended solutions. As shown in Figure 7, the spectra of silk fibroin in the mixtures show a negative peak between 210 and 220 nm, and a positive peak between 195 and 200 nm, characteristic features of beta sheet conformations [42]. This indicates that silk fibroin structure changes toward beta sheet conformation due to the interaction with collagen.

## 3. Discussion

Direct mixing of collagen and silk fibroin solutions resulted in phase separation, possibly due to the different pH values of silk fibroin solution (with pH of 7.17) and collagen solution (with pH of 2.74). The low collagen solution pH causes the protonation of carboxyl groups on silk fibroin to their non-ionic form, and amine groups to their cationic form. This is along with increasing the hydrophobicity of uncharged carboxyl groups with subsequent induction of silk fibroin morphological changes in the solution from the spherical micelles to nanofibrils. Additionally, conformational transition from random coil to β-sheet may occur [43,44,45]. Using higher temperatures (50–60 °C) as a way to prompt the interactions of collagen with other proteins through denaturating collagen molecules [22] is not the aim of this study, since the aim is the preservation of the collagen’s native structure. Hence, collagen and RSF solutions were mixed, using calcium salt ions of the ternary solvent for inducing electrostatic interaction between biopolymer chains.

The chaotropic behavior of divalent calcium ions, under a phenomenon called “salting in”, causes more ion-protein interactions than protein-protein interactions in the blend system, yielding a homogenous mixture [46]. The stability of the mixtures in the single-phase region is due to the quasi-equilibrium between oppositely-charged proteins (collagen and fibroin) and the introduction of a ternary solvent containing high amounts of salt which acts both as the third component of the phase diagram and a simple electrolyte.

In a system containing oppositely-charged proteins (considering their polyelectrolyte nature), increasing the ionic strength through adding salt to some extent, enhances the attraction between the oppositely-charged residues and causes a transition of an overcharged polyelectrolyte complex (PEC) to a neutral or uncharged complex [47,48]. Moreover, calcium ions with a radius of 4.1 Å in the hydrated state fit well to the distance of ~14 Å between adjacent triple helical molecules of collagen, probably interacting with the negatively-charged Asp or Glu side chains, forming salt bridges, and increasing the ionic strength of the solution [49,50,51].

**Figure 8:**
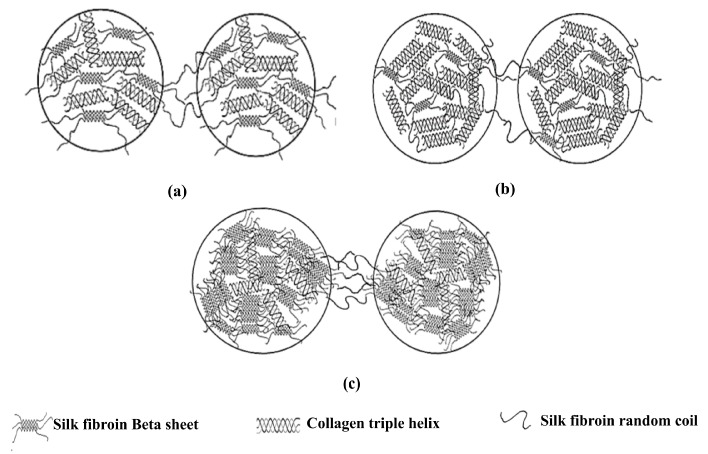
Schematic diagram of a hypothesized model for protein conformational changes in the two adjacent coacervate aggregates of collagen/RSF mixtures after dialysis with different starting ratios of: (**a**) Col/RSF: 75/25; (**b**) Col/RSF: 50/50; and (**c**) Col/RSF: 25/75.

Slow salt diffusion through the dialysis procedure changes the phase behavior of mixtures and causes coacervation or precipitation. Based on the results obtained from optical microscope images (Figure 3) and ζ potential analysis of the mixtures after dialysis, and considering the influence of proteins conformational structures on the aggregate formation, we hypothesized a model for mixtures with different ratios of collagen/silk fibroin (Figure 8).

In the mixtures containing collagen, it has been proved that the counterions were released to the media upon complexation with collagen [52]. Hence, in this research, at low ionic strength, the water and counterions may be released to the media upon the formation of hydrophobic and hydrogen bonds between the SF and collagen chains. Moreover, according to previous studies on silk fibroin mixtures [53], beta sheet conformation of silk fibroin showed by CD and FTIR analysis in this research may be due to the fact that upon complexation with collagen, silk fibroin chains may use collagen chains as a mold plate to stretch themselves. Through the process of the nucleation-dependent aggregation mechanism, once the beta sheet nucleus is formed, further growth of beta sheet units and beta sheet aggregation [54] will occur (Figure 8c).

On the other hand, the replacement of salt ions by water molecules (hydration) during dialysis causes the assembly of free collagen molecules, which probably happened in the mixtures with higher amounts of collagen (Figure 8b). Due to the specific hydroxyproline (Hyp) groups of collagen chains, the water molecules order around and between the collagen chains in a way that the triple helixes’ organization forms a crystal packing. In these crystals, the triple helixes are bridged by ordered water molecules, causing the collagen assembly that induces collagen molecules aggregation or fibrillogenesis [55].

Overall, the characterization methods used in this study are simple techniques for primary assessment of phase behavior in collagen/silk fibroin blends. Further investigation can be done on ternary phase diagram of collagen/RSF/ternary solvent in order to obtain a boundary of miscibility/coacervate/precipitate, through application of other methods such as turbidimetry and potentiometric titration. Additionally, the size, shape, and inner composition characteristics of the precipitates can be further studied by small angle X-ray technique (SAXS) or WAXS (wide angle X-ray).

## 4. Materials and Methods

### 4.1. Sample Preparation

Collagen solution was prepared by dissolving type I bovine collagen (Bovine Achilles tendon, Sigma-Aldrich, St. Louis, Missouri, USA) in 0.5 mol/L acetic acid to the final concentration of 0.5% (*w*/*v* %) and stirring with high speed Turrax (T25D, IKA^®^, Janke and Kunkel IKA-Labortechnik, Staufen, Germany) at 10,000 rpm and 4 °C for 2–3 h.

*B. mori* silk fibroin was prepared by initially degumming by boiling silkworm cocoons in Na_2_CO_3_ solution at 1 g/L for 30 min at 85 °C. This procedure was repeated two times and, finally, the cocoons were boiled in distilled water for 30 min in order to separate the glue-like sericin from fibroin. After adequate washing with distilled water, the obtained silk fibroin fibers were dried at room temperature. Finally, silk fibroin fibers were milled to facilitate their dissolution process. Silk fibroin was dissolved in a solution of ternary solvent containing CaCl_2_:CH_3_CH_2_OH:H_2_O (1:2:8 molar ratio), at 85 °C in order to obtain the final concentration of 0.5% (*w*/*v* %). The fibroin solution was dialyzed against distilled water for 72 h.

### 4.2. Ternary Phase Diagram and Blend Preparation

Considering the key role of salt ions for inducing the ionic interactions between protein chains in solvent, the ternary phase diagram of collagen, RSF and ternary solvent at 4 °C (in order to prevent collagen denaturation) was analyzed. Aiming at obtaining different Col/RSF ratios of 100/0, 75/25, 50/50, 25/75 and 0/100, collagen/RSF/ternary solvent blends were prepared by selecting the relevant points in single-phase region of ternary phase diagram. Identifying volume fractions of each component (X_component_ = V_component_/V_total_), the single-phase blends were prepared, at 4 °C. Later, the blended solutions were dialyzed against distilled water for three days. The resultant solutions were dried in polystyrene dishes at room temperature to obtain the blended films in the solid state.

### 4.3. Miscibility and ζ-Potential Analysis

In order to assess the miscibility, the viscosity behavior of mixtures in the single-phase region of ternary phase diagram, was analyzed at 25 ± 0.1 °C by Ubbelohde capillary viscometer (NCU Laboratory, Toruń, Poland). According to the intended blend ratios, different mass fractions of each polymer solution have been mixed. The intrinsic viscosity and the viscosity interaction parameter of each polymer solution as well as the ternary systems (collagen/RSF/ternary solvent) were obtained and used to estimate the miscibility of polymer mixtures through classical dilution method. Thus, each solution was prepared and diluted with NaCl (0.1 mol/L) to yield lower concentrations. The relative viscosities of blends were obtained by dividing solutions flow times by the value found for pure solvent (NaCl 0.1 mol/L). The intrinsic viscosity, the interaction parameter and Huggins coefficient values were determined according to the Huggins equation using solutions of several concentrations.

The values of experimental interaction parameter for all the blends, using diluted regime with solutions of 5 concentrations, were obtained from the plot of *η_sp_*/*c* vs *c* (mass concentration, in g/mL) using Equation (1):

(1) $$\frac{{\left(\eta_{sp}\right)}_{m}}{c_{m}}={\left[\eta \right]}_{m}^{exp}+b_{m}^{exp}c_{m}$$

where (*η_sp_*)/*c* is the reduced viscosity, $b_{m}^{exp}\;$ is the experimental viscosity interaction parameter of polymer mixture, ${\left[\eta \right]}_{m}^{exp}$ is the experimental intrinsic viscosity of the polymer blends, and $c_{m}$ is the total concentration of solution.

The ideal values in this study were determined according to the Krigbaum and Wall [28], and Garcia et al. [29] techniques. The ideal interaction parameter of $b_{m}^{id}$ have been determined by Krigbaum and Wall through Equation (2):

(2) $$b_{m}^{id*}=b_{A}w_{A}^{2}+b_{B}w_{B}^{2}+2b_{AB}^{id}w_{A}w_{B}$$

where $w_{A}$ and $w_{B}$ are the weight fractions of polymers A and B, respectively, and $b_{A}$ and $b_{B}$ are the interaction parameters of each individual polymer which were obtained from the slope of the plots of the reduced viscosity versus concentration. $b_{AB}$, the interspecific interaction parameter that was obtained by Equation (3):

(3) $$b_{AB}^{id}=b_{A}^{1/2}b_{B}^{1/2}$$

The ideal interaction parameter by Garcia et al. [29], was calculated through Equation (4):

(4) $$b_{m}^{id**}=b_{A}w_{A}^{2}+b_{B}w_{B}^{2}$$

The polymer mixture is miscible if $\Delta b_{m}=b_{m}^{exp}-b_{m}^{id}\;\geq \;0\;$ and immiscible if $\Delta b_{m}=b_{m}^{exp}-b_{m}^{id}\;\geq \;0$.

This viscosimetry analysis was done for all the mixtures after dialysis procedure. Thus, the solutions after dialysis have been passed through the gas and vacuum filters. The remaining solutions after filtration have been passed through two calibrated markers of the Ubbelhode viscometer, and the time has been measured.

The ζ-potential analysis was done for all solutions before and after dialysis through measuring the electrophoretic mobility by ZetaPALS (Brookhaven Instruments Corporation, Holtsville, NY, USA), as the temperature was maintained at 25 °C.

### 4.4. Light Stereoscopic Magnifier Microscope

The blend mixtures after dialysis procedure were observed and optical images were collected from the Leica EC3 stereo microscope equipped with Leica LAS software (Leica, Wetzlar, Germany). The mixtures in their containers were placed on a stage with a dark base and the pictures of the blend solutions were captured using the digital camera zoomed onto the solutions as closely as possible.

### 4.5. Structure of Collagen/Silk Fibroin Blended Solutions and Films after Dialysis

The structures of blended solutions after dialysis, were analyzed by circular dichroism (CD) using a J-815 (Jasco, Tokyo, Japan) spectrometer. Far-UV CD spectra were recorded between 190 and 260 nm using a 1 mm path length cuvette. CD spectra were acquired with a scanning speed of 100 nm/min, integration time of 1 s, and using a bandwidth of 1 nm. The spectra were averaged over eight scans and corrected by subtraction of the buffer signal. Spectra of silk fibroin in the mixtures were obtained by subtraction of pure collagen (Col/RSF 100/0 blend) spectrum from the mixture spectra. The results are expressed as the mean residue ellipticity ϴ_MRW_, defined by Equation (5):

ϴ_MRW_ = ϴobs(0.1MRW)/(lc) (5)

where ϴobs is the observed ellipticity (mdeg), MRW is the mean residue weight (g/mol), c is the concentration (mg/mL), l is the light path length (cm), and ϴ_MRW_ is the mean residue ellipticity (deg.cm²/dmol). MRW was calculated from data (MW and number of aminoacids) in the UniProt database as 76.1 for silk fibroin and 94.9 for collagen.

The structures of blended films were assessed by scanning electron microscopy (SEM), contact angle measurements, Fourier transform infrared spectroscopy (Perkin-Elmer 2000 FTIR spectrometer, Hopkinton, MA, USA), and differential scanning calorimetry (Setaram DSC 131, Caluire-et-Cuire, France).

For scanning electron microscopy (SEM), samples were coated with an Au/Pd thin film, by sputtering, using an SPI module sputter coater. The SEM analysis was performed using a high resolution (Schottky) environmental scanning electron microscope (FEI Quanta 400 FEG ESEM, Hillsboro, OR, USA). In order to analyze surface hydrophobicity, contact angle measurements were done using a digital imaging capture system (OCA 15, DataPhysics Instruments GmbH, Filderstadt, Germany). For this reason, the sessile drop method with distilled water at 25 °C was used and the contact angle was calculated using software version SCA 20. Thermal analysis of the prepared films has been performed using a Setaram DSC 131, from 25–450 °C at a scan rate of 10 °C/min with nitrogen flow of 50 mL/min. Moreover, FTIR analysis were carried out in the spectral range of 400–4000 cm^−1^ using a Perkin Elmer FTIR spectrophotometer model 2000, equipped with an ATR diamond cell accessory.

## 5. Conclusions

In this work, we studied the influence of salt ions on the phase behavior of collagen and silk fibroin mixtures. The results showed that ternary solvent containing calcium ions, as the third component in the ternary phase diagram, has a significant effect on the miscibility of mixtures. Its influence on implementing net charge density close to zero, making an almost electroneutral blend solution, was confirmed through the analysis of the observed ζ potential values. In such a system, the chains of collagen and silk fibroin should be in their most individual separated state with a high concentration of salt ions being placed among their chains. Moreover, the viscosity analysis reaffirmed the influence of ternary solvent to obtain high miscibility for all the blends. However, the maximum values were obtained for the mixture with the highest collagen ratio, due to the high ionic strength resulting from the higher amount of salt used in this mixture.

Removal of salt after the dialysis procedure yielded to complex coacervations (precipitation) with positive charge densities, demonstrating the importance of protein charge density and conformational structure. The protein-protein coacervate aggregates were formed as the oppositely charged protein chains got closer to each other and, after primary weak electrostatic interaction, the hydrogen and hydrophobic bonds were formed, leading to silk fibroin conformational changes from random coil to beta sheet. The conformational change of silk fibroin was reassured through CD spectra analysis of the solution after dialysis, as well as SEM, contact angle, DSC, and FTIR assessment of formed films after drying of dialyzed solutions.

Considering the physico-chemical changes of blended films, analyzed by SEM, contact angle, and DSC, it can be stated that the obtained blended films have tunable properties by varying the blend ratio. Overall, such preparation procedure for collagen/silk fibroin blends is a promising method for engineering of protein-protein interactions, which is important for the development of a wide range of biopharmaceutical applications of these materials, from drug delivery, to wound healing and tissue engineering.

## Figures and Tables

**Table 1:** ζ potential of collagen/RSF blends.

Sample	Zeta Potential (mV)
Collagen solution in acetic acid (0.5%)	39.06
Col/RSF: 75/25	1.41
Col/RSF: 50/50	3.12
Col/RSF: 25/75	−0.497

**Table 2:** Theoretical (by Krigbaum and Wall [28] and Garcia et al. [29] methods) and experimental values for pure collagen, pure silk fibroin and the mixtures.

W_Collagen_ (0.5%)	${\left[\eta \right]}_{m}^{exp}$ (dL/g)	${\left[\eta \right]}_{m}^{id}$ (dL/g)	∆[*η*]*_m_*	$b_{m}^{exp}$ (dL/g)^2^	$b_{m}^{id^{*}}$ (dL/g)^2^	∆b_m_*	$b_{m}^{id^{**}}$ (dL/g)^2^	∆b_m_**
1 (W_silk fibroin_:0)	2.48			38.44				
0.75	6.47	2.06	4.41	72.41	30.04	42.37	21.64	50.77
0.5	3.54	1.94	1.6	75.91	20.87	55.04	9.67	66.24
0.25	1.78	1.23	0.55	39.54	10.94	28.6	2.54	37.0
0 (W_silk fibroin_:1)	0.81			0.2433				

$b_{m}^{id^{*}}$: determined according to Krigbaum and Wall [28]; $b_{m}^{id^{**}}$: determined according to Garcia et al. [29].

**Table 3:** ζ potential of collagen/RSF blends after dialysis (the ratios are those before dialysis).

Sample	Zeta Potential (mV)
Silk fibroin after dialysis	−5.96
Col/RSF: 75/25 (after dialysis)	10.26
Col/RSF:50/50 (after dialysis)	9.03
Col/RSF:25/75 (after dialysis)	5.36

**Table 4:** Passing time (s) of the collagen/RSF solutions after filtrations (all the ratios are those before dialysis).

Dialysis Days	Col/RSF:75/25	Col/RSF:50/50	Col/RSF: 25/75	Water
Day 1	49.22 (s)	50.94 (s)	51.38 (s)	46.58 (s)
Day 2	48.59 (s)	49.32 (s)	49.95 (s)	46.58 (s)
Day 3	47.21 (s)	48.26 (s)	49.65 (s)	46.58 (s)

**Table 5:** The FTIR band assignments of collagen/RSF blends.

	Wavenumber (cm^−1^)		
	Amide I	Amide II	Amide III
Collagen	1634	1551	1238
Col/RSF: 75/25	1626	1531	1236
Col/RSF: 50/50	1644	1531	1237
Col/RSF: 25/75	1623	1525	1234
RSF	1644	1531	1237
